# Training for Supervisors to Improve Sustainable Employment of Employees with a work Disability: A Longitudinal Effect and Process Evaluation from an Intervention Study with Matched Controls

**DOI:** 10.1007/s10926-023-10118-2

**Published:** 2023-05-30

**Authors:** Rosanne Schaap, Pieter Coenen, Wim Zwinkels, Marianne de Wolff, Astrid Hazelzet, Johannes Anema

**Affiliations:** 1 Department of Public and Occupational Health, Amsterdam UMC, Amsterdam, The Netherlands; 2 Sustainable Productivity and Employability, Netherlands Organization for Applied Scientific Research TNO, Leiden, The Netherlands; 3 Epsilon Research, Leiden, The Netherlands

**Keywords:** Employees, Work disability, Sustainable Employment, Supervisors, Effect evaluation

## Abstract

**Purpose:**

Supervisors play a crucial role in sustainable employment of employees with a work disability. The ‘Mentorwijs’ (literal translation: Mentorwise) training was developed to train supervisors in knowledge, attitudes and skills needed to guide these employees. This study evaluated the effect of ‘Mentorwijs’ on employees’ employment and supervisors’ behavioral outcomes.

**Methods:**

Register- and questionnaire data were obtained from 73 employees and 1,526 matched controls to measure employment (≥ 1/month, ≥ 12 h/week and ≥ 3 consecutive months (≥ 1 h/month)) during a 12-month follow-up period. Questionnaire data were obtained from 127 supervisors who followed the ‘Mentorwijs’ training, to assess their knowledge, self-efficacy, intention to adopt and applied behaviors.

**Results:**

Employment for ≥ 1 h/month did not significantly improve after 3 (β = 0.05; CI=-0.07-0.16), 6 (β = 0.07; CI=-0.04-0.18), 9 (β = 0.08; CI=-0.02-0.18) and 12 (β = 0.01; CI=-0.08-0.10) months among employees whose supervisors followed ‘Mentorwijs’ compared to those who did not. Significant effects were found after 8 months (β = 0.11; CI = 0.01–0.21). Comparable effects were found for employment ≥ 12 hour/week and ≥ 3 consecutive months (≥ 1 hour/month). Supervisors’ knowledge and self-efficacy significantly improved as a result of ‘Mentorwijs’, but no effects were found for intention to adopt and applied behaviors.

**Conclusions:**

‘Mentorwijs’ is a promising training to improve the guidance of employees with a work disability. Further research is needed to examine how long-term effects of ‘Mentorwijs’ on employment can be sustained.

**Supplementary Information:**

The online version contains supplementary material available at 10.1007/s10926-023-10118-2.

## Background

Employees with a work disability face more difficulties to maintain employed, as opposed to those without a work disability [1]. Employees with a work disability could be hampered from finding or maintaining employed due to long-term illness, a disorder or disability, including (mild) intellectual disabilities, psychological frailty, physical disabilities, (very) low level of education and/or learning delay [2]. In the Netherlands, around 800 thousand people between 15 and 65 years indicated in 2019 that they faced difficulties to find and perform work due to a work disability [1]. Their unemployment rates are twice as high as in the general population [3]. Therefore, sustainable employment – defined as the ability to make a valuable contribution through work, while learning skills, maintaining good health and well-being throughout the working life [4] – remains a challenge among employees with a work disability.

Ample research indicates that supervisors play a crucial role in sustainable employment of employees with a work disability [5–11]. Barriers to remain employed were, for example, a lack of support from supervisors and colleagues and a lack of work accommodations [5]. Supervisors can reduce these barriers by establishing a supportive environment, promoting acceptance and inclusion of employees with a disability and enabling workplace accommodations. Other barriers were feeling incompetent, overqualified to execute work tasks or a lack of opportunities to learn new skills [5, 9]. Supervisors can reduce these barriers by giving appropriate feedback, providing clear task instructions and facilitating a work climate wherein employees can perform work tasks at their own pace and can learn from mistakes. However, to change behaviors and take away barriers, supervisors need specific knowledge, attitudes and skills for the guidance of employees with a work disability. They need to understand that employees with a work disability may have, for example, a lower work pace, than employees without a work disability [2]. Furthermore, some supervisors tend to take the role of a care provider, hindering employees to develop themselves. In such circumstances, it could be more important for supervisors not to focus on the disability and limitations, but on the competences and qualities of employees [2]. Based on these findings, it is likely that training supervisors in the guidance of employees with a work disability can improve their sustainable employability.

Previous research on training supervisors in the guidance of employees show that such trainings could lead to earlier return to work and reduced sick leave among employees, compared to employees whose supervisor was not trained (yet) [12, 13]. However, these studies focus on the general working population. ‘Mentorwijs’ (literal translation: Mentorwise) is a training developed to improve the guidance of supervisors of specifically employees with a work disability [2]. Evidence on the effectiveness of ‘Mentorwijs’ is, however, lacking. Also, more insight is needed on which employee and supervisors’ characteristics enhance or decrease the effectiveness of ‘Mentorwijs’, and how the implementation of this training proceeds. Based on these research gaps the aims of this study were to investigate (1) the effect of ‘Mentorwijs’ on sustainable employment of employees with a work disability, (2) the extent to which this effectiveness is affected by characteristics of employees and supervisors, (3) the effect of ‘Mentorwijs’ on supervisor guidance and (4) the implementation process of ‘Mentorwijs’.

## Methods

### Study Design

We conducted an intervention study that consisted of an effect and process evaluation among employees with a work disability (and a matched controls comparison group) and their supervisors who followed ‘Mentorwijs’. The effect evaluation among employees (i.e. aim 1 and 2) were conducted using questionnaire data completed by employees and register data from Statistics Netherlands. The effect and process evaluation among supervisors (i.e. aim 3 and 4) were conducted by the use of questionnaire data that were completed by supervisors. The Medical Ethics Committee of Amsterdam UMC (location VUmc) decided that the Medical Research Involving Human Subjects Act does not apply to this study (reference no. 2019.239). All participants who participated in this study provided informed consent. This study was registered in the Dutch Trial Register (Trial NL7901, 2019) [14]. The Consolidated Standards of Reporting Trials (CONSORT) was used as guideline to report this study [15].

### Intervention

‘Mentorwijs’ aims to develop and strengthen knowledge, attitudes, and skills of supervisors who guide employees with a work disability. A central element of the training is to strengthen self-efficacy, meaning that supervisors develop confidence that they have the knowledge and skills to adequately guide employees with a work disability. Furthermore, supervisors are trained on how to consider the work disability, while also taking the employee seriously and let them fully participate in a team where they can be equal to regular employees in the company. A more detailed description of the development and theoretical background of the intervention has been published elsewhere [2], but the specific goals and sub-goals of the training are described in Table 1. ‘Mentorwijs’ focuses on supervisors that guide employees with a work disability on a daily basis, as supervisors give work instructions and monitor the execution of work tasks. ‘Mentorwijs’ is a relatively short and practical training that consists of five weekly meetings of 2.5 h, each with specific learning objectives. The training was provided by Dutch municipalities and was free of charge for supervisors and involved organizations. Between 8 and 18 supervisors were expected to participate in each training. Each training was provided by two trainers who worked in a municipal organization. These organizations have the duty to enact the Participation Act (Participatiewet, in Dutch) which aims to help people with a disability to find a job, maintain employed and to support employers by wage subsidies, job coaches, trial placements or other forms of (financial) support. Supervisors enrolled in different ways for the training; through their employer or on an individual basis directly at the municipal organizations. The trainers did not need specific education to be able to provide the training, but were experienced trainers in the field of work and social security and were trained to provide the ‘Mentorwijs’ training. Homogeneity across training sites was assured by a train-the-trainer programme and a handbook of ‘Mentorwijs’. During the training there was variation between theoretical and practical work forms, where providing knowledge to supervisors was alternated with practical exercises to apply new knowledge. Methods that were applied in the training varied from lecturers, group discussions, case presentations, and role playing with ample opportunity for interaction between supervisors. Supervisors could bring up questions and cases from their daily practice, and worked preferably in different companies so they could share and exchange experiences with each other.

**Table 1:** Goals and sub-goals of ‘Mentorwijs’

Goal	Sub-goals
**Knowledge**: learning about work disabilities and how to deal with these disabilities	Knowledge about: • Various (common) psychological disabilities • Possible work adjustments for these disabilities • Support that can be offered by different stakeholders from municipalities (e.g. job coaches) • Which questions you can and may ask the employee prior to employment to gain insight into the employees’ qualities and limitations • Different leadership styles and which of these styles match the wishes and needs of an employee • Communication techniques (listening, summarizing, asking open questions)
**Attitude**: teaching how to maintain an open and involved attitude that increases the autonomy of employees	• Accept that employees have limitations to take into account, but not to overprotect • Have affinity with employees and wanting to take time to enhance work participation • Want to ensure that the employee enjoys going to work: feels heard, feels included • Being open to signals that indicate the employee is not doing well and ask about this in a positive way that is safe for the employee • Pay attention to possible frictions between employees with disabilities and regular employees: take initiative to discuss this in time • Pay attention to clarity and involvement of employees • Have an open and involved attitude towards the employee, without taking the role of a care provider • Make sure employees feel that you (are open to) listen to them • Have good observations skills without judgement
**Skills**: teaching specific skills regarding work and communication	Being able to: • Translate limitations into work adaptations: supervisors know how limitations affect daily functioning at the workplace, what kind of support employees need, which tasks employees can perform and which work adaptations are possible and needed • Create development opportunities for the employee, for example by organizing their work in a certain way • Use a transformational leadership style: motivate and encourage the employee in a respectful and honest manner • Find challenges for the employee, for example by letting the employee do other work tasks • Create support in the workplace/being able to deal with resistance • Observe/(timely) identify problems and being able to solve them, conflict management • Disseminate information about the employee to colleagues (in coordination with the employee) • Work together with external parties such as counselors from the municipality • Have a learning orientation: willing and able to exchange experiences and knowledge with others • Being a point of contact in the workplace for employees and colleagues • Identify and apply techniques for observing employees: being able to observe employees and recognize different competencies • Provide feedback in a constructive manner, use feedback to reduce resistance and to discuss the behavior of the employee • Identify which style of leadership or communication technique matches an employee • Contribute to improve the employee’s functioning and thereby create added value for the company

### Recruitment

‘Mentorwijs’ is implemented by different municipal organizations in the Netherlands. A total of 164 supervisors who guide employees with a work disability and signed up to follow ‘Mentorwijs’ between May 2019 and January 2021 were invited to participate in this study. Supervisors worked in different organizations in the Netherlands in the regions Rivierenland, Helmond-De Peel and Foodvalley, that employ employees with a work disability in sheltered workplaces and/or in the regular labor market. At the start of the training, researchers informed all 164 supervisors about the aim and methods of the study, and thereafter invited supervisors to participate in this study. If they agreed to participate, they provided informed consent and were asked to complete a baseline questionnaire at the start of the training. The follow-up questionnaires were completed online. Supervisors were also asked to help recruit employees with a work disability that they guided at the workplace. For every supervisor we aimed to recruit at least one employee with a work disability they guide at the workplace. However, it is unclear how many employees were invited to participate in this study. Supervisors asked their employees with a work disability whether the researchers could visit their workplace and to inform them about the study. After employees signed informed consent they were asked to complete a short questionnaire to, among other things, obtain information to identify employees in register data.

### Questionnaire data-collection Among Employees

Baseline questionnaires were completed by employees with a work disability between the start (T0) and completion of the training (T1), as employees were recruited through their supervisor who already started with the training. The questionnaire provided information on general characteristics of employees, type of work, type of work disability, work ability (i.e. based on the work ability index) [16] and work satisfaction.

### Register data-collection Among Employees

We used register data to gain more knowledge on sustainable employment of employees with a work disability whose supervisors participated in ‘Mentorwijs’ and from a matched control group of employees whose supervisors did not participate in ‘Mentorwijs’. Register data from Statistics Netherlands (CBS) on employment were available before and after the end of the training and were calculated on a monthly basis, up to 12 months. Primary outcome measures for sustainable employment were 1) being employed for at least 1 hour per month, 2) being employed for at least 12 hours per week, and 3) being employed for at least 3 consecutive months (≥ 1 hour/month). Secondary outcome measures for those in employment were type of contract, number of working hours per week and wage per hour. Also background characteristics of employees, job characteristics and employment and social security history were available from Statistics Netherlands.

### Intervention and Control Group of Employees with a work Disability

Register data was used to match the ‘Mentorwijs’ group to a similar group of employees. Therefore we selected employees in similar regions for Foodvalley (Stedendriehoek & Noord-West Veluwe), Rivierenland (Noord-Oost Brabant) and Helmond-De Peel (Noord-Limburg) and collected personal and current job characteristics as well as information on individual employment and social security history.

In the regions were ‘Mentorwijs’ was provided to supervisors we did not have an overview of which employees have a supervisor who did or did not follow the training. Therefore, employees in the control group were selected from other, comparable, regions as the ones in the intervention group, to make sure that employees were not guided by a supervisor who followed ‘Mentorwijs’. We matched on the following characteristics: sex, age, region, educational level, ethnical background, work history in 12 months before intervention, number of years in current job, unemployment or social assistance benefit as main income during at least 1 month in 12 months before intervention, sickness or disability benefits as main income during at least 1 month in 12 months before intervention, temporary contract, sector of economic activity, total number of employees of the employer, indicator semi-sheltered sector (i.e. sheltered workplace) and wage level. We used propensity score matching (nearest neighbor) with common support, because exact matching would have leaded to an additional loss of 20 ‘Mentorwijs’ employees that could not be matched.

### Questionnaire data-collection Among Supervisors

Self-reported questionnaires were used to obtain data on the effect and process of ‘Mentorwijs’ among supervisors who followed the training (i.e. aim 3 and 4). Questionnaires provided information on the personal and work characteristics of supervisors and outcome and process measures. Questionnaires were completed before the training (T0), directly after the training (T1) and 3 and 6 months after the end of the training (T2 & T3). Outcome measures for the effect evaluation were 1) determinants for behavior - i.e. knowledge regarding employees with a work disability and the supervision of this group and self-efficacy regarding the supervision of employees with a work disability, 2) intention to adopt behaviors regarding the supervision of employees with a work disability, and 3) the extent to which behaviors regarding the guidance of employees with a work disability were applied. Self-efficacy, intention to adopt and applied behaviors were, in accordance to the training, divided into attitudes and skills. For example, an item to measure attitude was that we asked supervisors whether they have self-efficacy, intention to adopt and actually applied an open and involved attitude towards employees with a disability. An item to measure skills was, for example, that we asked supervisors whether they have self-efficacy, intention to adopt and actually applied a supporting environment at the workplace for employees with a work disability.

Reliability and validity was not tested, but items for each outcome measure were based on the ‘Mentorwijs’ theoretical handbook [2]. The items in the questionnaire were aligned to the defined objectives and expected results in this theoretical handbook. Process measures (only measured after the training – T1) focused on factors that could affect the implementation of the training in practice: 1) dose delivered – i.e. to what extent was the intervention implemented as planned, 2) dose received – i.e. number of meetings followed, 3) satisfaction towards the training, 4) extra time spend on the guidance of employees with a disability, 5) and contextual factors on the level of the supervisor and organization, which were based on an existing instrument to measure determinants of innovations [17].

### Statistical Analysis

For aim 1 we applied a difference-in-difference estimation to the matched sample in Stata 14, which allowed us to estimate the causal effect of ‘Mentorwijs’. The difference-in-difference estimation together with matching corrects for potential pre-treatment differences between the ‘Mentorwijs’ and control group. A similar approach has been followed by De Graaf-Zijl et al (2020) [18]. In the analysis, every person in the control group is weighted according to their propensity score. The use of difference-in-difference techniques is only allowed if there is a common trend between Mentorwijs and the control group prior to the intervention. Tests showed that a placebo effect of Mentorwijs 6 months before the actual start of the intervention was not statistically significant for any of the outcome measures. This implies that the common trend hypothesis for using the difference-in-difference design has not been violated.

The model specification is:

$${Y}_{it}= {\tau }_{t}+\sum _{t=1}^{12}{\beta }_{t}{MW}_{i}{T}_{it}+\sum _{t=-16}^{12}{\gamma }_{t}{T}_{it}+{\epsilon}_{it}$$

Where i is the individual employee and t calendar time. *Y*_*it*_ is the outcome of interest (employment status) for individual i in month t. Individuals have to be employed in month 0. Month 1 is the month of the end of the intervention or fictional intervention in case i belongs to the control group. ${\tau}_{t}$ are quarterly calendar time dummies for each quarter, and can capture business cycle and other time calendar time effects. ${MW}_{i}$ is an indicator taking the value 1 if the individual is in the ‘Mentorwijs’ group. ${T}_{it}$ are time dummies representing the month compared to the start of (fictive) treatment. ${\epsilon}_{it}$ is the error term. ${\beta }_{t}$ and ${\gamma }_{t}$ are parameters, and is the effect of analysis time. Note that controls do not necessarily have to start in the same month as ‘Mentorwijs’ cases, meaning that calendar time and analysis time can differ. ${\beta }_{t}$ is the parameter of interest, the estimate of the effect of ‘Mentorwijs’. The beta is the difference in the change of the outcome between the intervention and control group in month t, with respect to the baseline measurement. For aim 2 we used the same main model but with interaction effects for subgroups.

For aim 3 we used mixed modeling in SPSS statistics 26 to estimate the change after ‘Mentorwijs’ on all outcomes measures among supervisors, wherein time was used as a categorical independent variable and T0 was used as the reference category (model 1). This technique deals better with missing data than generalized estimation equations (GEE), and considers that repeated measurements are correlated [19]. In a second model we tested for the following possible confounders 1) demographics (i.e. age, educational level and sex), 2) number of years of experience with the guidance of employees with a work disability, 3) number of years employed at current employer, 4) company size, 5) number of employees they guide at the workplace and 6) number of employees with a work disability they guide at the workplace. Only confounders that changed the beta of the independent variable (i.e. time) with more than 10% were added to the model (model 2). In both models we estimated Beta coefficients (B) and 95% confidence intervals (CI). For aim 4 process evaluation data were analyzed using descriptive statistics (i.e. mean (SD) and percentage).

## Results

### Participants

**Fig. 1:**
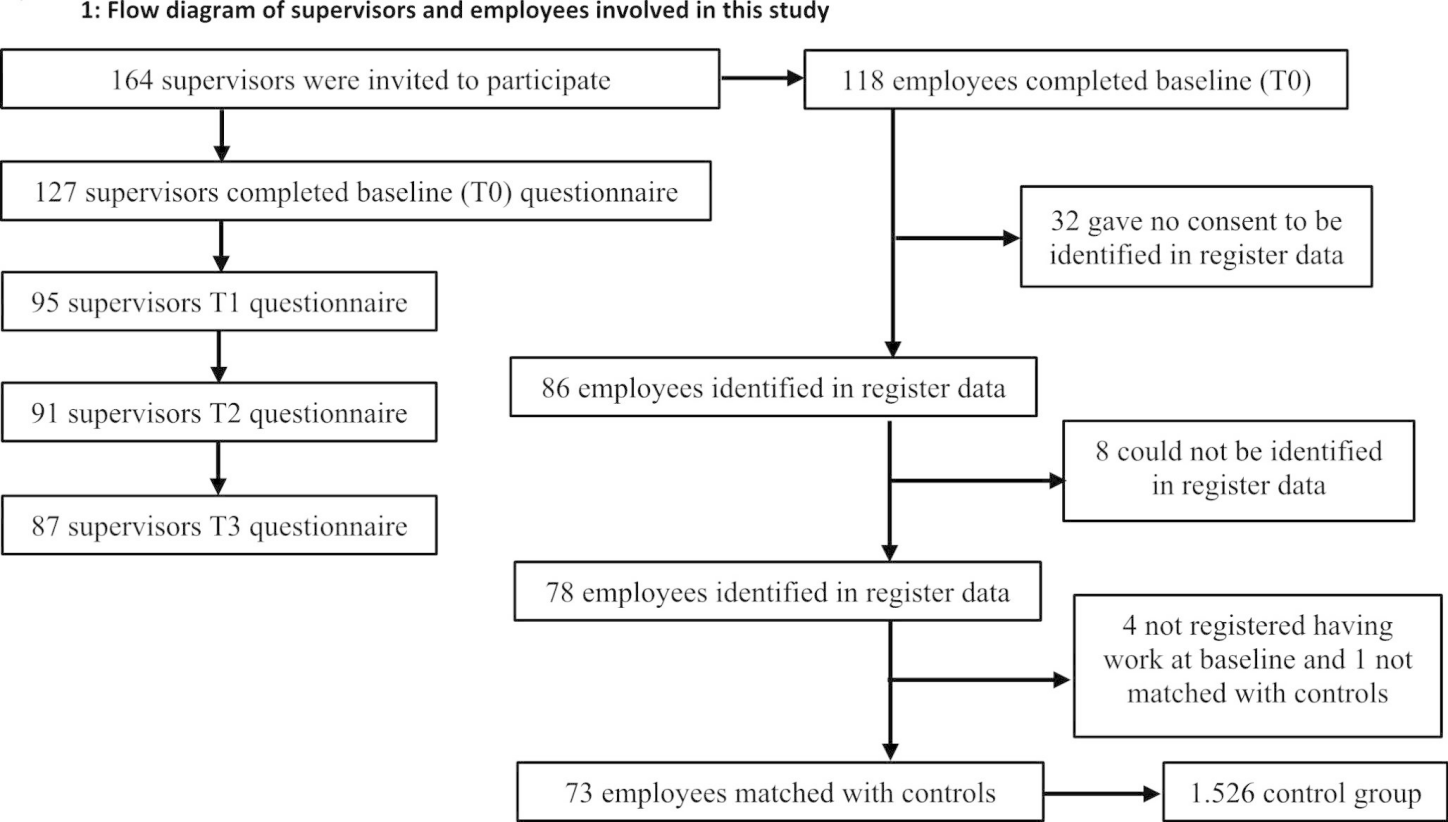
Flow diagram of supervisors and employees involved in this study

We included 127 supervisors that followed ‘Mentorwijs’ and 118 employees with a work disability who were guided by these supervisors. Not every employee gave consent to be identified in the register data and not every employee could be identified in the register data. Therefore, register data were collected from 78 employees. Four employees were excluded from matching with controls, as they were not registered as having a job at baseline. One employee could not be matched with controls. In the end, 73 employees were matched with 1.526 controls. Figure 1 shows the flow diagram of the selection process of supervisors and employees in this study.

### Characteristics of Employees with a work Disability

The results in the baseline questionnaire showed that employees in the intervention group had very different occupations ranging from industrial work (26%), service related (17%), transport related (6%), administrative work (6%), specialized work – e.g. ICT, draftsmen (4%), or in agriculture or landscaping (41%). All type of work disabilities were represented in the intervention group, 22% had a mild intellectual disability, 18% a psychological disability, 35% a physical disability, 25% a low level of education/learning delay and for 19% the work disability was unknown. The work ability was often good or excellent (62%) and the mean work ability in relation to job demands was 6.0 (SD 0.9) on a scale from 2 to 10. The majority was satisfied or very satisfied (81%) with their work. In Table 2, characteristics of employees in the intervention and control group in register data are presented.

**Table 2:** Characteristics of employees with a work disability in register data

Characteristics	N = 73; %/mean (SD) intervention group register data	N = 1526; %/mean (SD) control group register data
**Sex**		
Male	77%	75%
Female	23%	25%
**Mean Age**	44.8 (13.4)	43.5 (13.8)
**Educational level**		
Low	53%	53%
High	27%	28%
Unknown	20%	19%
**Ethnic background**		
Western/no migration background	86%	90%
Non-western/migration background	14%	10%
**Region (Intervention vs. control)**		
Foodvalley / Stedendriehoek & Noord-West Veluwe	32%	29%
Rivierenland / Noord-Oost Brabant	27%	28%
Helmond-De Peel / Noord-Limburg	41%	43%
**Type of contract**		
Permanent contract	56%	55%
Temporary contract	44%	45%
**Wage per hour**		
≤ 13 euro’s per hour	22%	17%
> 13 euro’s per hour	78%	83%
**Number of months work before intervention**		
0–10 months	21%	24%
11–12 months	79%	76%
**Numbers of years in current job** 0–1 year	11%	11%
2–5 years	41%	39%
> 5 years	48%	50%
**Social welfare benefit 12 months before intervention**		
Yes	14%	17%
No	86%	83%
**Work disability benefit 12 months before intervention**		
Yes	95%	94%
No	5%	6%
**Sector**		
Government	71%	67%
Non-governmental	29%	33%
**Type of workplace**		
Sheltered workplace	51%	53%
Regular workplace	49%	47%
**Company size**		
< 250 employees	25%	27%
≥ 250 employees	75%	73%

### Characteristics of Supervisors Guiding Employees with a work Disability

The study sample of supervisors mostly consisted of males (71%) (Table 3). Most supervisors worked in a governmental organization (29%) and had on average 4.7 (4.9) years of experience with the guidance of employees with a work disability. The majority (67%) guided less than 10 employees with a work disability. See Table 3 for more information on the characteristics of supervisors.

**Table 3:** Characteristics of supervisors

Baseline characteristics supervisors	N = 95 %/mean (SD)
**Sex**	
Male	71%
Female	29%
**Mean age**	44.8 (10.8)
**Educational level**	
Low	26%
Middle	37%
High	35%
Unknown	2%
**Number of hours working per week**	35.9 (5.9)
**Company size**	
0-250 employees (SME)	47%
> 250 employees	51%
Unknown	2%
**Type of organization**	
Agriculture and landscaping	15%
Industry and construction	14%
Transport and trade	13%
Service and hospitality	18%
Education	7%
Health care and welfare	4%
Government	29%
**Number of years employed at current employer**	9.93 (9.8)
**Number of years of experience with guidance of employees**	4.69 (4.9)
**Number of employees guiding at work**	
1–10	39%
> 10	48%
Unknown	13%
**Number of employees with a work disability guiding at work**	
1–10	62%
> 10	31%
Unknown	7%

### Effect of ‘Mentorwijs’ on Sustainable Employment of Employees with a work Disability (aim 1)

**Fig. 2:**
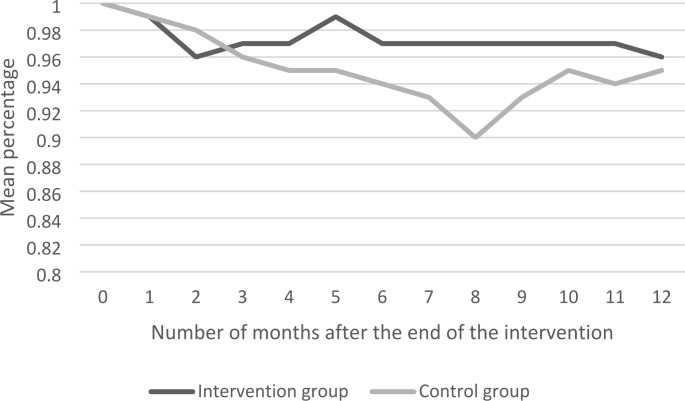
Employed ≥ 1 h per month

**Fig. 3:**
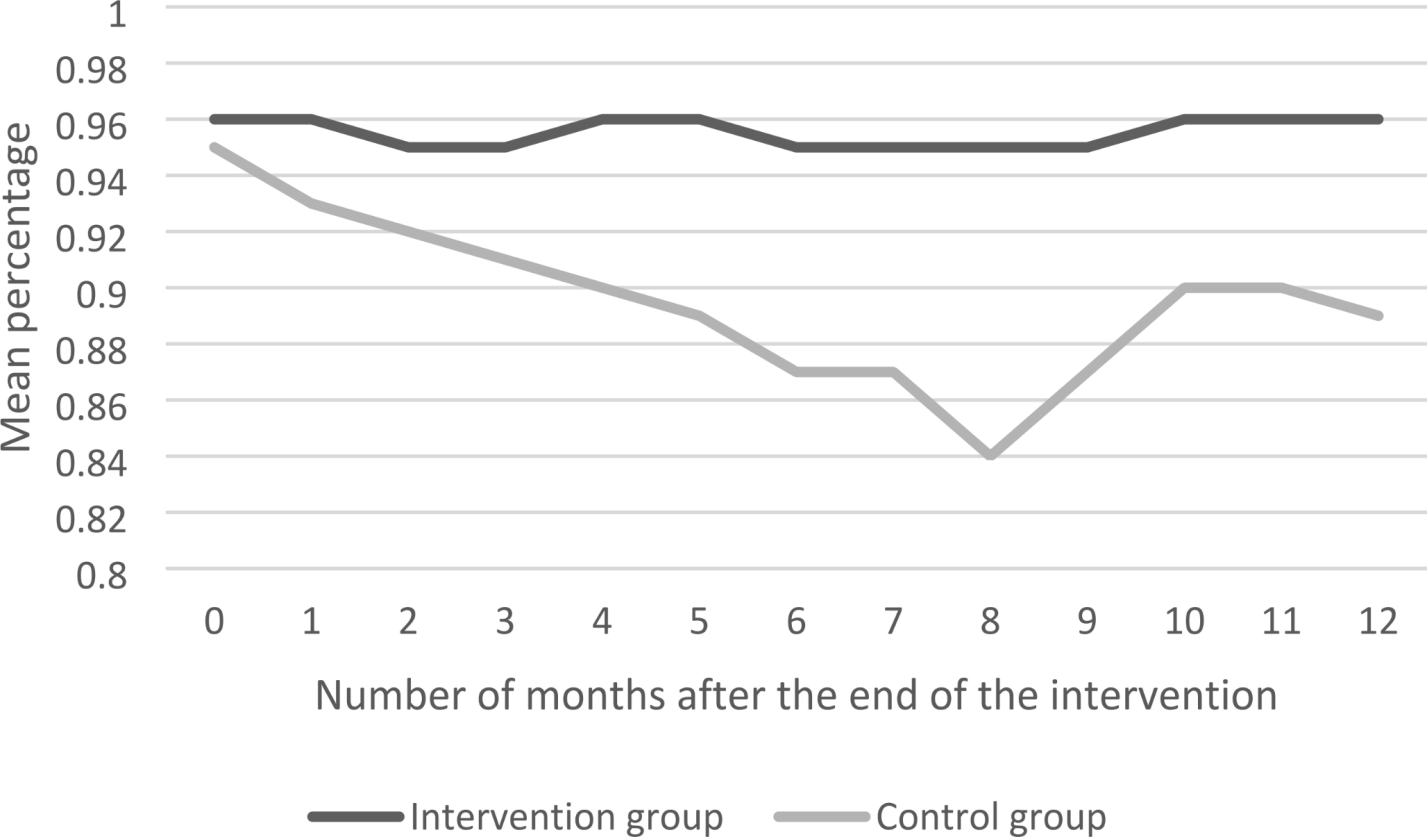
Employed ≥ 12 h per week

**Fig. 4:**
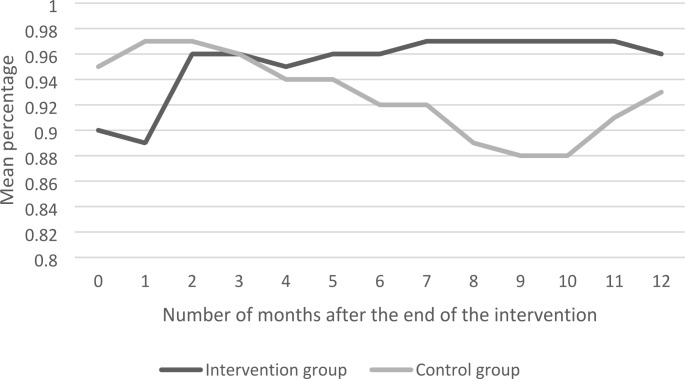
Employed for 3 consecutive months (≥ 1 h/month)

Table 4 shows the intervention effects (i.e. the betas) at the end of the training (T1), and 3 (T2), 6 (T3), 9 (T4) and 12 months (T5) after the end of the training, with effects of other months shown in Supplementary file 1. The beta is the difference in the change of the outcome being employed between the intervention and control group at a certain time point (T), compared to the baseline measurement. In Figs. 2–5 the same intervention effects are shown for all months for the intervention and control group, for the outcomes being employed (≥ 1 h/month), for being employed 12 h per week or more and for being employed for 3 consecutive months (≥ 1 h/month). The results in Table 4 show that the intervention group is more often employed (≥ 1 h/month) after 3 (β = 0.05; CI=-0.07-0.16), 6 (β = 0.07; CI=-0.04-0.18), 9 (β = 0.08; CI=-0.02-0.18) and 12 (β = 0.01; CI=-0.08-0.10) months than the control group, but these differences were not significant. Hence, the betas show that, although not statistically significant, there is a tendency of a decrease in the amount of employees being employed being larger in the control group than in the intervention group. However, differences between the intervention and control group could also be due to sampling variability, as the results in Fig. 2 show that for being employed (≥ 1 h/month) only a statistical significant difference was found after 8 months (betas reported in the supplementary file 1). For being employed 12 h per week the same results were found, which is shown in Fig. 3. For being employed for 3 consecutive months (≥ 1 h/month) no significant differences were found at any point in time, which is shown in Fig. 4. Moreover, Figs. 2–4 also show that the outcomes on employment were relatively stable in the intervention group and relatively erratic in the control group. For the outcome measure having a temporary contract, the proportion of employees with a temporary contract decreased in the intervention and control group, but no significant differences between groups were found. Regarding the number of hours employees work per week, the results in Table 4 show that after 6 months the intervention group works significantly more hours than the control group (β = 1.70; CI = 0.29–3.11). However, after 12 months these differences attenuated (β = 0.11; CI=-1.36-1.59). The results for wage per hour increases in both the intervention and control group, but differences were not significant.

**Table 4:** Difference-in-Difference analysis outcome measures employees at the end of the training (T1), 3 (T2), 6 (T3), 9 (T4) and 12 months (T5) after the end of the training

Primary and secondary outcome measures employees	N intervention	N control	Mean (SD)/% intervention	Mean (SD)/% control	β	95%-CI	P-value
**Employed ≥ 1 hours/month**							
T1 (end of the training)	73	1526	100%	100%			
T2 (3 months after the training)	73	1526	97%	96%	0.05	-0.07 to 0.16	0.437
T3 (6 months after the training)	73	1526	97%	94%	0.07	-0.04 to 0.18	0.202
T4 (9 months after the training)	73	1526	97%	93%	0.08	-0.02 to 0.18	0.130
T5 (12 months after the training)	73	1526	96%	95%	0.01	-0.08 to 0.10	0.834
**Employed ≥ 12 hours/week**							
T1	73	1526	96%	95%			
T2	73	1526	95%	91%	0.08	-0.03 to 0.19	0.154
T3	73	1526	95%	87%	0.09	-0.03 to 0.19	0.119
T4	73	1526	95%	87%	0.07	-0.04 to 0.17	0.203
T5	73	1526	96%	89%	0.04	-0.07 to 0.14	0.470
**Employed for 3 consecutive months (≥ 1 hours/month)**							
T1	73	1526	90%	95%			
T2	73	1526	96%	96%	0.02	-0.10 to 0.14	0.750
T3	73	1526	96%	92%	0.07	-0.06 to 0.20	0.311
T4	73	1526	97%	88%	0.10	-0.02 to 0.22	0.106
T5	73	1526	96%	93%	0.02	-0.09 to 0.13	0.726
**Temporary contract**							
T1	73	1526	37%	41%			
T2	71	1467	34%	39%	-0.01	-0.17 to 0.14	0.847
T3	71	1437	35%	31%	0.07	-0.10 to 0.24	0.411
T4	71	1424	32%	26%	0.13	-0.03 to 0.30	0.119
T5	70	1437	27%	24%	0.01	-0.14 to 0.16	0.915
**Number of hours working per week**							
T1	73	1526	30.19 (8.5)	28.97 (9.6)			
T2	71	1467	30.11 (8.5)	28.86 (9.5)	0.47	-1.00 to 1.95	0.529
T3	71	1437	30.70 (8.0)	28.65 (10.0)	1.70	0.29 to 3.11	0.018
T4	71	1424	30.72 (8.0)	29.05 (9.6)	0.70	-0.49 to 1.90	0.249
T5	70	1437	31.82 (6.9)	29.19 (9.7)	0.11	-1.36 to 1.59	0.881
**Wage per hour**							
T1	73	1526	11.30 (1.4)	12.06 (3.6)			
T2	71	1467	11.35 (1.4)	12.14 (3.4)	-0.03	-0.19 to 0.12	0.662
T3	71	1437	11.44 (1.4)	12.18 (3.5)	-0.09	-0.25 to 0.06	0.220
T4	71	1424	11.59 (1.4)	12.09 (3.2)	0.14	-0.08 to 0.35	0.211
T5	70	1437	11.68 (1.7)	12.19 (3.4)	0.24	-0.01 to 0.50	0.064

### Characteristics Affecting the Effectiveness of ‘Mentorwijs’ (aim 2)

The results show that the effect of ‘Mentorwijs’ tended to be stronger among employees with a temporary contract and with a social welfare benefit 12 months before the end of the intervention, as opposed to employees without a temporary contract and a social welfare benefit (Supplementary file 2). The betas in supplementary file 2 are presented for one subgroup. For instance, the results in supplementary file 2 show that the betas for employees that had a social welfare benefit (i.e. within one subgroup) were positive after 3 (β = 0.28), 6 (β = 0.29), 9 (β = 0.27), and 12 (β = 0.27) months. This means that the effect of the training in the intervention group is stronger among employees that had a social welfare benefit, and that the effect of the training is weaker among employees without a social welfare benefit. Moreover, the effect of ‘Mentorwijs’ also tended to be stronger among employees that have a supervisor that guides less than 10 employees with a work disability. Conversely, the results show that the effect of ‘Mentorwijs’ tended to be weaker among employees in the governmental sector, working in an organization with more than 250 employees, working in sheltered workplaces and with a work disability benefit 12 months before the end of the intervention.

### Effect of ‘Mentorwijs’ on Supervisor Guidance of Employees with a work Disability (aim 3)

Table 5 shows that knowledge and self-efficacy for attitudes and skills of supervisors significantly improved between T0, and all follow-up moments after the training. Improvements were mainly between T0 and T1, and then remained stable over time. For intention to adopt attitudes significant effects were also found between T0 and all follow-up moments. However, for intention to adopt and applied attitudes and skills no significant effects were found.

**Table 5:** Linear regression showing the effect of all outcome measures at the supervisor level before (TO), directly after (T1) and 3 (T2) and 6 months (T3) after the end of the training

			Model 1: Unadjusted			Model 2: Adjusted model		
Outcome measures	N	Mean (SD)	β	95%-CI	P-value	β	95%-CI	P-value
**Knowledge**								
T0 (reference category)	127	3.35 (0.79)						
T1	95	4.14 (0.63)	0.77	0.62 to 0.92	0.000	0.77*	0.62 to 0.92*	0.000*
T2	91	4.22 (0.64)	0.87	0.72 to 1.03	0.000	0.87*	0.72 to 1.03*	0.000*
T3	87	4.23 (0.62)	0.89	0.74 to 1.05	0.000	0.89*	0.74 to 1.05*	0.000*
**Self-efficacy**								
Attitude								
T0 (reference category)	127	4.23 (0.64)						
T1	95	4.52 (0.54)	0.29	0.15 to 0.43	0.000	0.29*	0.15 to 0.43*	0.000*
T2	91	4.56 (0.58)	0.32	0.18 to 0.47	0.000	0.32*	0.18 to 0.47*	0.000*
T3	87	4.59 (0.54)	0.36	0.22 to 0.51	0.000	0.36*	0.22 to 0.51*	0.000*
Skills								
T0 (reference category)	127	3.84 (0.68)						
T1	95	4.33 (0.53)	0.49	0.34 to 0.63	0.000	0.49*	0.34 to 0.63*	0.000*
T2	91	4.32 (0.61)	0.48	0.36 to 0.63	0.000	0.48*	0.36 to 0.63*	0.000*
T3	87	4.32 (0.66)	0.49	0.34 to 0.64	0.000	0.49*	0.34 to 0.64*	0.000*
**Intention to adopt behavior**								
Attitude								
T0 (reference category)	127	4.60 (0.42)						
T1	95	4.75 (0.35)	0.14	0.06 to 0.22	0.001	0.14**	0.05 to 0.23**	0.003**
T2	91	4.71 (0.43)	0.19	0.02 to 0.18	0.016	0.10**	0.01 to 0.20**	0.028**
T3	87	4.66 (0.49)	0.07	-0.02 to 0.15	0.111	0.09**	0.00 to 0.18**	0.049**
Skills								
T0 (reference category)	127	4.26 (0.61)						
T1	95	4.29 (0.64)	0.03	-0.08 to 0.14	0.615	0.06***	-0.08 to 0.20***	0.382***
T2	91	4.28 (0.59)	0.02	-0.09 to 0.13	0.708	0.03***	-0.11 to 0.17***	0.669***
T3	87	4.28 (0.64)	0.04	-0.07 to 0.15	0.465	0.03***	-0.11 to 0.16***	0.683***
**Apply behaviors**								
Attitude								
T0 (reference category)	121	3.64 (0.71)						
T1	93	3.77 (0.75)	0.08	-0.07 to 0.24	0.277	0.04****	-0.14 to 0.22****	0.638****
T2	86	3.75 (0.84)	0.09	-0.07 to 0.25	0.253	-0.04****	-0.23 to 0.14****	0.645****
T3	83	3.88 (0.82)	0.24	0.08 to 0.40	0.004	0.17****	-0.01 to 0.35****	0.067****
Skills								
T0 (reference category)	117							
T1	91	4.44 (0.58)	0.08	-0.06 to 0.22	0.264	0.03****	-0.15 to 0.20****	0.769****
T2	86	4.30 (0.72)	-0.05	-0.19 to 0.10	0.507	-0.09****	-0.27 to 0.08****	0.293****
T3	83	4.36 (0.72)	0.017	-0.13 to 0.16	0.817	-0.01****	-0.19 to 0.16****	0.890****

*no confounders changed the beta of the independent variable with more than 10%; **adjusted for number of years of experience; ***adjusted for number of years of experience, number of years employed at current employer, educational level, number of employees guiding at the workplace, number of employees with a work disability guiding at the workplace; **** adjusted for number of years of experience, number of years employed at current employer, educational level, number of employees they guide at the workplace, number of employees with a work disability they guide at the workplace, company size

### Implementation Process of ‘Mentorwijs’ (aim 4)

In this study a total of 19 ‘Mentorwijs’ trainings that each consisted of five meetings were evaluated. The intervention was delivered to groups, ranging from 5–18 supervisors in one training. The majority of the trainings (n = 14) took place at municipal organizations or at the workplaces of supervisors (Table 6). Five trainings took place online due to the Covid-19 Pandemic. Most supervisors (73%) participated in all 5 meetings of a single training and the training was on average evaluated as satisfying (mean satisfaction score ranging from 4.4 to 4.7 (on a scale from 1–5). Between 25 and 31% of the supervisors indicated they spend on average 4–7 hours extra time on the guidance of employees with a work disability after completion of the training. The majority of supervisors rated almost all contextual factors a high score. The supervisors rated feedback and formal endorsement from their own organization lower, as compared to other contextual factors.

**Table 6:** Process evaluation measures

Process evaluation outcomes		Mean (SD)/%
**Dose delivered**	Training at municipal organization or at workplace	74%
	Online training	26%
**Dose received**	Participated in 5 meetings of a training	73%
	Participated in 4 meetings of a training	18%
	Participated in 3 meetings of a training	7%
	Participated in 2 meetings of a training	0%
	Participated in 1 meeting	1%
**Extra time spent on guidance**	Guidance of employees takes more time (yes)	T1 = 25%
		T2 = 31%
		T3 = 26%
	Number of hours per week spent extra on guidance	T1 = 6.6 (8.3)
		T2 = 5.5 (4.8)
		T3 = 4.7 (3.9)
**Satisfaction**	Satisfaction in general	4.4 (0.6)
	Satisfaction meetings	4.4 (0.5)
	Satisfaction trainer(s)	4.7 (0.9)
	Satisfaction content of the training	4.4 (0.9)
	Satisfaction teaching methods of the training	4.4 (0.9)
	Satisfaction structure and duration of the training	4.4 (0.9)
**Contextual factors on supervisor and organizational level**	Outcome expectation: I expect ‘Mentorwijs’ to succeed in improving the employability of employees with a work disability	4.4 (0.8)
	Task perception: I consider it part of my job to apply what I have learned in the training to the guidance of employees	4.6 (0.7)
	Satisfaction employees: Employees are in general satisfied if I use what I have learned in the training	4.2 (0.9)
	Self-efficacy expectation: I am able to use what I have learned in the training in the guidance of employees	4.1 (0.6) ***
	Sufficient staff: There is sufficient staff in our organization to apply what I have learned in the training	4.1 (1.0)
	Financial resources: I receive sufficient financial resources from our organization to apply what I have learned in the training	4.2 (1.0)
	Time: I get enough time from our organization to apply what I learned in the training	4.4 (0.8)
	Feedback: In my organization there is regular discussion with employers about what I have learned in the training and how it can improve the guidance of employees and how to implement this in the guidance	3.3 (1.2)
	Formal endorsement: Formal agreements in the organizational policies have been made by the management and/or employer about guiding employees corresponding to what supervisors have learned in the training	Yes = 31%
		No = 28%
		I don’t know = 41%

*Scale 1–5; 1 = very unsatisfied, 5 = very satisfied; **Scale 1–5; 1 = totally disagree, 5 = totally agree; ***Scale 1–5; 1 = most definitely not, 5 = most definitely yes

## Discussion

On employee level, ‘Mentorwijs’ significantly improved outcomes on employment after 8 months. ‘Mentorwijs’ tended to have a positive effect on the sustainable employability of employees with a work disability, as can be obtained from Figs. 2–4. In these figures, the ‘Mentorwijs’ group outcomes on employment showed a relatively stable tendency over time, as compared to the control group, and thereby prevented early drop-out from work. However, only significant differences between intervention and control group were found 8 months after the end of the training. Employees for whom the training tended to be more effective were employed with a temporary contract, had a social welfare benefit, and a supervisor that guides less than 10 employees with a work disability. In contrast, employees for whom the training tended to be less effective were employed in the governmental sector, sheltered workplaces, larger organizations and had a work disability benefit. On supervisor level ‘Mentorwijs’ significantly improved knowledge and self-efficacy, but no effects were found on intention to adopt and applied behaviors. The process evaluation showed that supervisors were generally satisfied about the training, and most contextual factors that may affect implementation of ‘Mentorwijs’ scored relatively high.

### Interpretation of Findings Regarding Effects ‘Mentorwijs’ on Outcomes Sustainable Employment (aim 1)

In this study we found small effects of ‘Mentorwijs’ on sustainable employment. Significant effects for outcomes on employment were found after 8 months, but attenuated after 12 months. This is in line with another study that also found positive effects of a supervisor training on the short-term among employees [13]. Still, effects in this study are small and attenuate after 8 months, which could be explained by factors that lay outside the scope of ‘Mentorwijs’ and could not be adjusted for in this study. For instance, the type of contract could affect the extent to which supervisors apply the training to employees. Supervisors are more often inclined to invest in an employee with a permanent contract and facilitate workplace adjustments or offer training opportunities, as opposed to employees with a temporary contract [20]. This is, however, in contrast to our findings that the ‘Mentorwijs’ training was most effective among employees with a temporary contract. An explanation for this could be that more proximal factors within workplaces have a greater impact on sustainable employability than the guidance of supervisors. For example, temporary contracts for employees with a work disability are often not converted into a permanent contract [20]. Moreover, at 12 months follow-up there is a high probability that one-year temporary contracts have ended. This may explain the lack of differences between the intervention and control group after 12 months, as a training for supervisors most likely does not have a large influence on changing temporary contracts into permanent contracts. Furthermore, workplaces that are characterized by a very high level of job insecurity may result in feelings of anxiety and financial stress among employees [20]. Hence, having a supervisor who is more supportive may not be sufficient to improve employees’ sustainable employability. This is underlined by research showing that factors such as an open and safe organizational climate also play a role in the sustainable employability of employees with a work disability [21].

### Interpretation of Findings Regarding Characteristics Affecting the Effectiveness of ‘Mentorwijs’ (aim 2)

This study also showed that certain characteristics enhanced or decreased the effect of ‘Mentorwijs’ on sustainable employment. The training tended to be less effective among employees in larger organizations, possibly due to less attention for each individual employee in these type of organizations. In addition, there may also work other disadvantaged employees, such as older employees, that need additional support to remain employed [22]. In contrast, literature also shows that the employment of employees with a work disability is higher in larger organizations, as supervisors have more flexibility to support employees with a disability [23]. This could result in an improved job performance and employability, as supervisors can provide more appropriate accommodations [24]. The finding that the training tended to be less effective in larger organizations, is in line with our finding that the effect of the training tended to be more effective among employees that have a supervisor that guides less than 10 employees with a work disability. These employees might receive more personal attention and/or support from their supervisor. The training also tended to be less effective among employees that worked in the governmental sector or sheltered workplaces. This is striking, because the governmental sector has the highest share of organizations that employ people with a disability [25], and sheltered workplaces are especially created for employees with a work disability that are not able to work in the regular labor market. The effect of ‘Mentorwijs’ might be less effective, as in these type of workplaces more employees with severe disabilities could be employed which have a higher chance of dropping out of the labor market. Thereby, a supervisor training might not be sufficient to enhance the sustainable employability of employees with a work disability. The latter may also account for employees that had a work disability benefit, for whom the effect of the supervisor training also tended to be weaker. In contrast, the training tended to be more effective for employees that had a social welfare benefit. This group of employees could be less vulnerable and are often temporarily unemployed, as opposed to those with a work disability benefit, meaning there is higher chance that employees with a social welfare benefit improve their sustainable employability.

### Interpretation of Findings Regarding Effect ‘Mentorwijs’ on Behavioral Outcomes Supervisor (aim 3)

This study found that ‘Mentorwijs’ had positive effects on supervisor knowledge and self-efficacy. A systematic review and meta-analyses on training managers to support and understand the mental health of employees found similar results [26]. Although, just like in our study, this review highlighted that no information is available on the long-term effects of such trainings among supervisors. Furthermore, our training did not render any effects on intention to adopt and applied behaviors. The training is relatively short (i.e. 5 meetings over 5 weeks) which could be insufficient to change these behavioral outcomes. Moreover, some trainings took place online which could hamper the effectiveness of the training, as it may be more difficult for the trainers to notice non-verbal signals or to adequately respond to the needs of supervisors. A lack of effect on intention to adopt and applied behaviors may also may be because changes in behaviors for the guidance of employees with a work disability are difficult to measure. We based the items of the questionnaire on the theoretical handbook of ‘Mentorwijs’ [2]. However, the training also leaves plenty of room to respond to the needs of supervisors and to share experiences from practice. The latter were not measured in our questionnaire. Furthermore, supervisors already scored relatively high on (intention to) behaviors at baseline, and therefore placing a limitation on the potential improvement of these outcome measures. Alternatively, supervisors self-reported behaviors may reflect social desirability, resulting in more favorable reporting in the intention to adopt or applied behaviors.

### Interpretation of Findings Regarding Implementation Process of ‘Mentorwijs’ (aim 4)

Next to the methodological explanations described above, the extent to which supervisors can implement the training largely depends on contextual factors. The path from a training being perceived as helpful by a supervisor, to the ability and opportunity to implement their newly acquired knowledge, attitudes and skills in daily work settings, to employees noticing these changes, and also to measure changes among supervisors and employees is rather complex and difficult to intervene upon [27]. Contextual factors (such as support from managers, sufficient time and resources and organization’s climate and culture) may form barriers or facilitators along this pathway and also may have played a role in the lack of significant effects on employment outcome among employees. Researchers have argued that the organizational conditions or work environment are highly important to understand effects of a training in organizations [28, 29]. During the intervention and evaluation period organizational changes may have occurred that could impact the transfer of the supervisor training at the workplace. This type of information, such as the impact of the measures for covid-19, was not captured, and therefore remains uncertain. By using an intervention and matched control group for the effect evaluation among employees we could not match, or sufficiently control for, organizational changes in our statistical analyses. Such changes, and other relevant confounding factors may play a role in the implementation of ‘Mentorwijs’ and should therefore be considered in future research. Furthermore, the extent to which the implementation of a training is embedded in organizational policies is also important. Organizational policies regarding employment of employees with a work disability facilitates the sustainable employment of these employees [23, 30]. These type of policies may provide supervisors more time and resources for the guidance of employees with a work disability. The process evaluation in this study showed that about one third of the supervisors spend on average 4–7 h more time on the guidance of employees with a disability after completion of the training. The extent to which companies provide supervisors extra time to spend on the guidance could play a role in the exact amount of hours supervisors can spend on doing this. The process evaluation also showed that supervisors scored less positive on two factors, namely feedback and formal endorsement. These factors, which are not part of ‘Mentorwijs’, could hamper the implementation of ‘Mentorwijs’ in practice, and may explain the lack of effects on intention to adopt or applied behaviors.

### Strengths & Limitations

To our knowledge this is the first study that evaluates the effectiveness of a supervisor training to improve the guidance of employees with a work disability on the level of supervisors and employees, with a long-term follow-up period among employees. However, this study also contained several methodological limitations. First, the selection of employees with work disabilities was done by supervisors and might have resulted in selection bias. Supervisors may have selected a “better” employee to participate in this study. This might have biased the effects of ‘Mentorwijs’, in which the training may be less effective than our results suggest. Second, a small sample size of employees could also have biased the results and may have contributed to only finding significant effects at 8 months. Third, the control group of employees with a work disability was identified in other regions than the intervention group, and the allocation to the intervention group was not randomized. To address this limitation we used a propensity score matching method to achieve optimal comparability between the groups in terms of primary outcomes measures and additional matching criteria [31]. This allowed to control for major confounding variables, such as age, gender and employment characteristics. Although, this does not exclude that unobserved or unmeasurable factors, such as type of work disability, organizational culture, and HR-policies, might have influenced our results and may have reduced the comparability between the intervention and control group. Fourth, selection bias might also have occurred in the group of supervisors that were followed over time. Supervisors already scored relatively high on certain behavioral outcomes. This may reflect that supervisors who participated in this study already had a more positive attitude towards the guidance of employees with a work disability, and therefore placing a limitation on the potential improvement of these measures. Another limitation is that the evaluation among supervisors did not contain a control group, which cannot totally exclude that intervention effects were caused by elements other than the training itself. Moreover, recall bias may also have occurred as supervisors were asked to complete the questionnaire four times within a short period of times between measurements, meaning that supervisors may have remembered the questions in the questionnaire and could fill in the same answers. Although, this does not account for the effect evaluation among employees by using register data.

### Implications for Research and Practice

This study showed that the effects of a supervisor training on employee and supervisor level are mixed and difficult to capture. Taking into account the methodological limitations of this study, there is a need for a higher quality study design to examine the effectiveness of ‘Mentorwijs’. A larger sample size and randomization of employees and supervisors could avoid the main limitations of this study – i.e. selection bias and the influence of unobserved or unmeasurable factors. Furthermore, qualitative research is needed to gain more insight into the experiences of supervisors with the training itself, but also what kind of elements (i.e. content and/or teaching methods) of the training were relevant for supervisors to implement at the workplace. Moreover, more research is also needed on organizational factors (e.g. feedback and formal endorsement) that enable supervisors to implement the training. For instance, research should be conducted on how organizational factors influence the guidance of employees with a work disability and how employers could be persuaded to implement factors that positively enhance the guidance, such as support from management and sufficient time and resources.

Training supervisors in the guidance of employees with a work disability is highly recommended, as the importance of their role in the organization is widely recognized [10, 21]. However, this study only found significant effects on knowledge and self-efficacy among supervisors, while effects on sustainable employment were only significant at 8 months and thereafter attenuated and became non-significant. As described above, the training was relatively short, thus to sustain effects we may need to think about a follow-up of the training or (monthly) return meetings. In these meetings supervisors can for example exchange experiences about the implementation of the training or further discuss certain aspects of the training. As was also described above, the effectiveness of the training is highly dependent on contextual factors. When employers do not make informed decisions on how these kind of interventions can be effectively implemented in organizations, possibly in combination with or as an addition to other interventions, the effects remain uncertain. Trainings, such as ‘Mentorwijs’ need to be integrated in organizational policies to reassure that supervisors have sufficient time and resources to implement their newly acquired knowledge, attitudes and skills. Considering the role of contextual factors (e.g. support from managers or resources) it would be useful to, in addition to ‘Mentorwijs’, also provide a training taking such organizational factors into account. Moreover, every organization may have other needs regarding the training of supervisors to improve the guidance of employees with a work disability. Therefore, effectiveness of trainings, such as ‘Mentorwijs’ could be improved by addressing the needs of an organization before the start of the training or adapting the training in consultation with employers. Lastly, HR or management of organizations should, next to offering trainings to supervisors, structurally strive for measures that improve the inclusion of employees with a disability, as this may also result in more employment opportunities and human resources practices for employability [32]. This is important, as solely implementing a supervisor training may not be enough to improve sustainable employment of employees.

## Conclusion

‘Mentorwijs’ is a promising training to improve the guidance of employees with a work disability. Small positive effects were found on the sustainable employability of employees, but effects attenuated in the long-term. Among supervisors the training mainly improved knowledge and self-efficacy. Further research is needed to examine whether these promising findings of ‘Mentorwijs’ can be replicated in studies with a larger sample size and reduced chance on selection bias. A follow-up of the training may be needed to also improve intention to adopt and applied attitudes and skills of supervisors and thereby the sustainable employability of employees on the longer term. Further research is also needed to examine how this intervention could be successfully implemented to increase the effectiveness for supervisors and employees, taking contextual factors into account.

### Electronic Supplementary Material

Below is the link to the electronic supplementary material.

Supplementary Material 1

Supplementary Material 2

## Data Availability

Questionnaire data are available from the corresponding author on reasonable request. Register data are available from Statistics Netherlands on reasonable request.
