# Supplementary material for: Training for Supervisors to Improve Sustainable Employment of Employees with a work Disability: A Longitudinal Effect and Process Evaluation from an Intervention Study with Matched Controls
Source: J Occup Rehabil. 2023 May 30;34(1):180–96. doi: 10.1007/s10926-023-10118-2 (PMC10227387; doi:10.1007/s10926-023-10118-2)
Supplement: Supplementary file 1 — Supplementary Material 1 [file 10926_2023_10118_MOESM1_ESM.docx]

**Supplementary file 1**

**Table 1. Difference-in-Difference analysis outcome measures employees 1-12 months after the end of the training**

| **Primary and secondary outcome measures employees** | **β** | **95%-CI** | **P-value** |
| --- | --- | --- | --- |
| **Employed≥1 hours/month**  1 month after the training  2 months after the training  3 months after the training (T2)  4 months after the training  5 months after the training  6 months after the training (T3)  7 months after the training  8 months after the training  9 months after the training (T4)  10 months after the training  11 months after the training  12 months after the training (T5) | 0.02  0.02  0.05  0.06  0.08  0.07  0.08  0.11  0.08  0.05  0.02  0.01 | -0.04 to 0.08  -0.09 to 0.13  -0.07 to 0.16  -0.06 to 0.17  -0.03 to 0.19  -0.04 to 0.18  -0.03 to 0.19  0.01 to 0.21  -0.02 to 0.18  -0.05 to 0.16  -0.08 to 0.13  -0.08 to 0.10 | 0.533  0.714  0.437  0.348  0.154  0.202  0.160  0.027  0.130  0.330  0.633  0.834 |
| **Employed ≥12 hours/week**  1 month after the training  2 months after the training  3 months after the training (T2)  4 months after the training  5 months after the training  6 months after the training (T3)  7 months after the training  8 months after the training  9 months after the training (T4)  10 months after the training  11 months after the training  12 months after the training (T5) | 0.04  0.08  0.08  0.11  0.08  0.09  0.09  0.10  0.07  0.05  0.04  0.04 | -0.03 to 0.10  -0.04 to 0.19  -0.03 to 0.20  -0.01 to 0.24  -0.03 to 0.19  -0.02 to 0.20  -0.02 to 0.21  0.00 to 0.21  -0.04 to 0.17  -0.06 to 0.16  -0.06 to 0.14  -0.07 to 0.14 | 0.311  0.187  0.154  0.067  0.148  0.119  0.124  0.047  0.203  0.327  0.452  0.470 |
| **Employed for 3 consecutive months (≥1 hours/month)**  1 month after the training  2 months after the training  3 months after the training (T2)  4 months after the training  5 months after the training  6 months after the training (T3)  7 months after the training  8 months after the training  9 months after the training (T4)  10 months after the training  11 months after the training  12 months after the training (T5) | -0.03  -0.00  0.02  0.04  0.05  0.07  0.10  0.10  0.10  0.11  0.04  0.02 | -0.09 to 0.02  -0.13 to 0.12  -0.10 to 0.14  -0.09 to 0.17  -0.08 to 0.18  -0.06 to 0.20  -0.03 to 0.24  -0.02 to 0.22  -0.02 to 0.22  -0.02 to 0.23  -0.07 to 0.16  -0.09 to 0.13 | 0.263  0.985  0.750  0.536  0.426  0.311  0.138  0.118  0.106  0.093  0.455  0.726 |
| **Temporary contract**  1 month after the training  2 months after the training  3 months after the training (T2)  4 months after the training  5 months after the training  6 months after the training (T3)  7 months after the training  8 months after the training  9 months after the training (T4)  10 months after the training  11 months after the training  12 months after the training (T5) | 0.03  -0.03  -0.01  0.01  0.07  0.07  0.09  0.12  0.13  0.12  0.03  0.01 | -0.01 to 0.08  -0.18 to 0.12  -0.17 to 0.14  -0.16 to 0.17  -0.09 to 0.24  -0.10 to 0.24  -0.09 to 0.26  -0.05 to 0.29  -0.03 to 0.30  -0.06 to 0.30  -0.12 to 0.18  -0.14 to 0.16 | 0.155  0.714  0.847  0.945  0.380  0.411  0.348  0.163  0.119  0.183  0.733  0.915 |
| **Number of hours working per week**  1 month after the training  2 months after the training  3 months after the training (T2)  4 months after the training  5 months after the training  6 months after the training (T3)  7 months after the training  8 months after the training  9 months after the training (T4)  10 months after the training  11 months after the training  12 months after the training (T5) | 0.66  0.86  0.47  1.29  1.55  1.70  1.73  0.36  0.70  0.55  -0.05  0.11 | -0.44 to 1.76  -0.36 to 2.08  -1.00 to 1.95  -0.04 to 2.62  0.07 to 3.03  0.29 to 3.11  0.22 to 3.24  -0.86 to 1.58  -0.49 to 1.90  -0.83 to 1.93  -1.48 to 1.38  -1.36 to 1.59 | 0.241  0.168  0.529  0.058  0.041  0.018  0.025  0.562  0.249  0.433  0.941  0.881 |
| **Wage per hour**  1 month after the training  2 months after the training  3 months after the training (T2)  4 months after the training  5 months after the training  6 months after the training (T3)  7 months after the training  8 months after the training  9 months after the training (T4)  10 months after the training  11 months after the training  12 months after the training (T5) | -0.11  0.03  -0.03  -0.03  0.04  -0.09  0.01  0.16  0.14  0.20  0.24  0.24 | -0.23 to 0.01  -0.11 to 0.17  -0.19 to 0.12  -0.16 to 0.09  -0.12 to 0.21  -0.25 to 0.06  -0.17 to 0.19  -0.01 to 0.34  -0.08 to 0.35  -0.01 to 0.42  0.01 to 0.48  -0.01 to 0.50 | 0.075  0.658  0.662  0.606  0.599  0.220  0.917  0.072  0.211  0.066  0.044  0.064 |
