# Supplementary material for: Training for Supervisors to Improve Sustainable Employment of Employees with a work Disability: A Longitudinal Effect and Process Evaluation from an Intervention Study with Matched Controls
Source: J Occup Rehabil. 2023 May 30;34(1):180–96. doi: 10.1007/s10926-023-10118-2 (PMC10227387; doi:10.1007/s10926-023-10118-2)
Supplement: Supplementary file 2 — Supplementary Material 2 [file 10926_2023_10118_MOESM2_ESM.docx]

**Supplementary file 2**

**Table 2. Difference-in-Difference analysis for being employed (≥1/month) stratified by characteristics of supervisors and employees 3 (T2), 6 (T3) and 12 months (T4) after the end of the training**

| **Employed (yes/no) stratified by characteristic of employees and supervisors** | **β** | **95%-CI** | **P-value** |
| --- | --- | --- | --- |
| **Employed and being a women**  T2  T3  T4  T5 | 0.04  0.04  0.04  -0.01 | -0.10 to 0.17  -0.10 to 0.17  -0.09 to 0.18  -0.10 to 0.07 | 0.574  0.574  0.544  0.746 |
| **Employed by age**  T2  T3  T4  T5 | 0.00  -0.00  -0.00  -0.00 | -0.00 to 0.01  -0.00 to 0.00  -0.00 to 0.00  -0.00 to 0.00 | 0.934  0.683  0.718  0.672 |
| **Employed and western**  T2  T3  T4  T5 | 0.07  0.07  0.07  0.12 | -0.22 to 0.36  -0.23 to 0.37  -0.23 to 0.37  -0.18 to 0.40 | 0.642  0.656  0.656  0.466 |
| **Employed and high educational level**  T2  T3  T4  T5 | 0.13  0.07  0.07  0.02 | -0.01 to 0.28  -0.08 to 0.22  -0.07 to 0.22  -0.10 to 0.15 | 0.073  0.363  0.325  0.720 |
| **Employed in region Rivierenland**  T2  T3  T4  T5 | 0.04  0.08  0.12  0.07 | -0.15 to 0.23  -0.11 to 0.27  -0.09 to 0.31  -0.13 to 0.27 | 0.658  0.416  0.244  0.826 |
| **Employed in region Helmond/De Peel**  T2  T3  T4  T5 | -0.12  -0.01  0.01  -0.02 | -0.26 to 0.02  -0.16 to 0.14  -0.15 to 0.18  -0.19 to 0.15 | 0.722  0.904  0.877  0.496 |
| **Employed in governmental sector**  T2  T3  T4  T5 | -0.14  -0.19  -0.07  -0.14 | -0.33 to 0.06  -0.37 to -0.00  -0.17 to 0.02  -0.33 to 0.05 | 0.166  0.044  0.120  0.137 |
| **Employees working in sheltered workplace**  T2  T3  T4  T5 | -0.21  -0.19  -0.17  -0.15 | -0.33 to -0.10  -0.32 to -0.06  -0.31 to -0.02  -0.30 to -0.01 | 0.000  0.005  0.023  0.040 |
| **Employed in organization ≥250 employees**  T2  T3  T4  T5 | -0.11  -0.17  -0.16  -0.20 | -0.30 to 0.08  -0.35 to 0.00  -0.34 to 0.02  -0.39 to -0.00 | 0.245  0.052  0.086  0.045 |
| **Employed for more than 5 years**  T2  T3  T4  T5 | -0.21  -0.19  -0.16  -0.12 | -0.31 to -0.10  -0.30 to -0.07  -0.29 to -0.04  -0.24 to 0.01 | 0.000  0.002  0.012  0.068 |
| **Employed with a temporary contract**  T2  T3  T4  T5 | 0.20  0.24  0.23  0.18 | 0.07 to 0.33  0.12 to 0.36  0.09 to 0.36  0.05 to 0.31 | 0.002  0.000  0.001  0.006 |
| **Employed with ≤13 euro’s wage per hour**  T2  T3  T4  T5 | -0.13  -0.05  -0.06  0.01 | -0.33 to 0.08  -0.26 to 0.16  -0.27 to 0.15  -0.18 to 0.19 | 0.220  0.647  0.590  0.922 |
| **Employed with a social welfare benefit at 12 months before intervention**  T2  T3  T4  T5 | 0.29  0.28  0.27  0.27 | 0.11 to 0.48  0.10 to 0.46  0.09 to 0.45  0.10 to 0.45 | 0.002  0.003  0.003  0.003 |
| **Employed with a work disability benefit at 12 months before intervention**  T2  T3  T4  T5 | -0.11  -0.08  -0.06  -0.06 | -0.18 to -0.04  -0.15 to -0.01  -0.14 to 0.01  -0.14 to 0.01 | 0.002  0.026  0.099  0.097 |
| **Employed by work disability**  T2  T3  T4  T5 | -0.01  -0.00  -0.01  -0.00 | -0.05 to 0.04  -0.05 to 0.04  -0.04 to 0.03  -0.04 to 0.03 | 0.765  0.826  0.737  0.896 |
| **Employed and with a bad or average work ability**  T2  T3  T4  T5 | 0.13  0.08  0.09  0.05 | 0.00 to 0.26  -0.05 to 0.21  -0.05 to 0.22  -0.07 to 0.17 | 0.046  0.227  0.195  0.411 |
| **Employed and satisfied/very satisfied about work**  T2  T3  T4  T5 | -0.04  0.04  0.04  0.02 | -0.19 to 0.10  -0.11 to 0.18  -0.09 to 0.18  -0.12 to 0.15 | 0.558  0.639  0.540  0.791 |
| **Employed with a mild intellectual disability**  T2  T3  T4  T5 | -0.09  -0.09  -0.09  -0.06 | -0.24 to 0.05  -0.24 to 0.05  -0.24 to 0.06  -0.21 to 0.08 | 0.194  0.211  0.232  0.378 |
| **Employed with a psychological disability**  T2  T3  T4  T5 | 0.03  -0.06  -0.06  -0.05 | -0.16 to 0.22  -0.26 to 0.14  -0.26 to 0.15  -0.25 to 0.16 | 0.749  0.540  0.584  0.648 |
| **Employed with a physical disability**  T2  T3  T4  T5 | 0.08  0.09  0.10  0.06 | -0.06 to 0.21  -0.05 to 0.22  -0.03 to 0.24  -0.06 to 0.18 | 0.255  0.198  0.136  0.359 |
| **Employed with a low level of education/learning delay**  T2  T3  T4  T5 | 0.01  0.00  -0.01  0.01 | -0.11 to 0.12  -0.11 to 0.11  -0.11 to 0.09  -0.08 to 0.11 | 0.893  0.999  0.859  0.786 |
| **Employed by age supervisor**  T2  T3  T4  T5 | 0.00  0.00  0.00  0.00 | -0.01 to 0.01  -0.01 to 0.01  -0.01 to 0.01  -0.01 to 0.01 | 0.805  0.639  0.621  0.977 |
| **Employed and women as supervisor**  T2  T3  T4  T5 | 0.14  0.04  0.02  -0.05 | -0.05 to 0.32  -0.16 to 0.24  -0.19 to 0.24  -0.23 to 0.13 | 0.154  0.721  0.858  0.600 |
| **Employed and supervisor with a high educational level**  T2  T3  T4  T5 | -0.05  -0.03  -0.02  0.02 | -0.17 to 0.07  -0.15 to 0.09  -0.14 to 0.10  -0.09 to 0.13 | 0.405  0.660  0.763  0.731 |
| **Employed and number of hours supervisor works**  T2  T3  T4  T5 | 0.00  0.01  0.01  0.01 | -0.02 to 0.03  -0.02 to 0.03  -0.02 to 0.03  -0.01 to 0.04 | 0.760  0.508  0.469  0.312 |
| **Employed and number of years supervisors employed at current employer**  T2  T3  T4  T5 | 0.00  0.00  0.00  0.00 | -0.00 to 0.01  -0.00 to 0.01  -0.00 to 0.01  -0.00 to 0.01 | 0.711  0.405  0.452  0.284 |
| **Employed and supervisors that guides <10 employees**  T2  T3  T4  T5 | 0.10  -0.01  -0.07  -0.10 | -0.08 to 0.27  -0.20 to 0.17  -0.24 to 0.10  -0.24 to 0.04 | 0.284  0.876  0.430  0.158 |
| **Employed and supervisor that guides <10 employees with a work disability**  T2  T3  T4  T5 | 0.19  0.14  0.10  0.08 | 0.03 to 0.34  -0.03 to 0.31  -0.05 to 0.26  -0.06 to 0.23 | 0.020  0.102  0.186  0.260 |
| **Employed and supervisor number of years of experience**  T2  T3  T4  T5 | -0.01  -0.01  -0.01  -0.01 | -0.02 to 0.00  -0.03 to 0.00  -0.03 to 0.00  -0.02 to 0.00 | 0.091  0.067  0.060  0.227 |
